# Cri du Chat syndrome

**DOI:** 10.1186/1750-1172-1-33

**Published:** 2006-09-05

**Authors:** Paola Cerruti Mainardi

**Affiliations:** 1 Paediatrics Department and Genetics Unit, S.Andrea Hospital, Vercelli, Italy

## Abstract

The Cri du Chat syndrome (CdCS) is a genetic disease resulting from a deletion of variable size occurring on the short arm of chromosome 5 (5p-). The incidence ranges from 1:15,000 to 1:50,000 live-born infants. The main clinical features are a high-pitched monochromatic cry, microcephaly, broad nasal bridge, epicanthal folds, micrognathia, abnormal dermatoglyphics, and severe psychomotor and mental retardation. Malformations, although not very frequent, may be present: cardiac, neurological and renal abnormalities, preauricular tags, syndactyly, hypospadias, and cryptorchidism. Molecular cytogenetic analysis has allowed a cytogenetic and phenotypic map of 5p to be defined, even if results from the studies reported up to now are not completely in agreement. Genotype-phenotype correlation studies showed a clinical and cytogenetic variability. The identification of phenotypic subsets associated with a specific size and type of deletion is of diagnostic and prognostic relevance. Specific growth and psychomotor development charts have been established. Two genes, Semaphorin F (*SEMAF*) and **δ**-catenin (*CTNND2*), which have been mapped to the "critical regions", are potentially involved in cerebral development and their deletion may be associated with mental retardation in CdCS patients. Deletion of the telomerase reverse transcriptase (*hTERT*) gene, localised to 5p15.33, could contribute to the phenotypic changes in CdCS. The critical regions were recently refined by using array comparative genomic hybridisation. The cat-like cry critical region was further narrowed using quantitative polymerase chain reaction (PCR) and three candidate genes were characterised in this region. The diagnosis is based on typical clinical manifestations. Karyotype analysis and, in doubtful cases, FISH analysis will confirm the diagnosis. There is no specific therapy for CdCS but early rehabilitative and educational interventions improve the prognosis and considerable progress has been made in the social adjustment of CdCS patients.

## Disease name/synonyms

Cri du Chat syndrome

5p deletion

## Definition

Cri du Chat Syndrome (CdCS) is a genetic disease resulting from a deletion of the short arm of chromosome 5 (5p-). Its clinical and cytogenetic aspects were first described by Lejeune *et al*. in 1963 [1]. The most important clinical features are a high-pitched cat-like cry (hence the name of the syndrome), distinct facial dysmorphism, microcephaly and severe psychomotor and mental retardation. The size of the deletion ranges from the entire short arm to the region 5p15 [2]. Simmons *et al*. reported a deletion size ranging from 5 to 40 Mb [3].

## Epidemiology

CdCS is a rare disease with an incidence ranging from 1:15,000 [4] to 1:50,000 [5] live-born infants. Niebuhr [5] found a prevalence of around 1:350 among over 6,000 mentally retarded people, Duarte *et al*. [6] found a prevalence of 1:305 among 916 patients attending genetic counselling services and analysed cytogenetically.

## Clinical description

**Figure 1:**
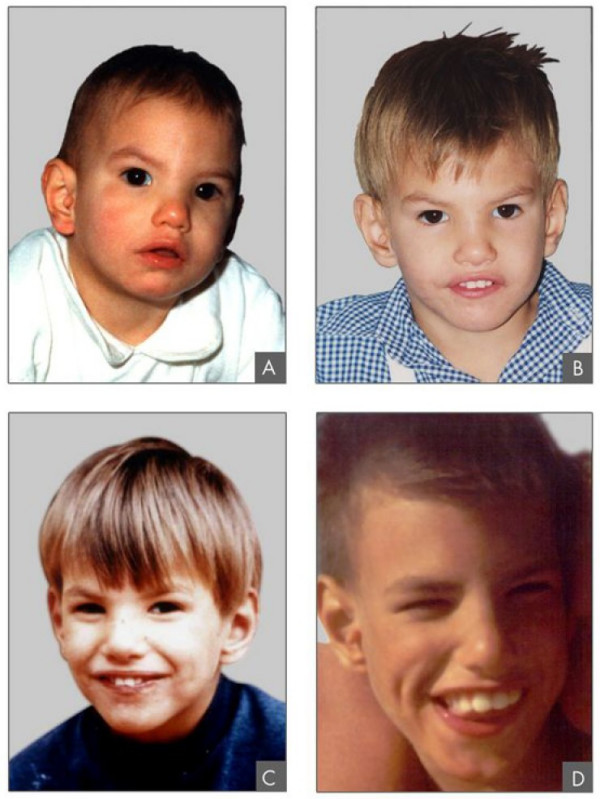
Clinical features of a patient with Cri du Chat syndrome at age of 8 months (A), 2 years (B), 4 years (C) and 9 years 6/12 (D).

The clinical features at birth are low weight (mean weight 2614 g), microcephaly (mean head circumference 31.8 cm), round face (83.5%), large nasal bridge (87.2%), hypertelorism (81.4%), epicanthal folds (90.2%), downward slanting palpebral fissures (56.9%), down-turned corners of the mouth (81.0%), low-set ears (69.8%), micrognathia (96,7%), abnormal dermatoglyphics (transverse flexion creases) (92%) and the typical cry (95.9%) [1,5,7-19] (percentages from the Italian CdCS Registry [19]) (Fig. 1A,B). Neonatal problems are asphyxia, cyanotic crises, impaired suction and hypotonia. Severe psychomotor retardation becomes evident during the first year of life. Malformations, although not very frequent, may be present: cardiac, neurological and renal abnormalities, preauricular tags, syndactyly, hypospadias, and cryptorchidism. Recurrent respiratory and intestinal infections are reported during the first years of life, although higher sensibility to infections is not reported [20].

The characteristic cat-like cry is probably due to anomalies of the larynx (small, narrow, diamond-shaped) and of the epiglottis (flabby, small, hypotonic), as well as to neurological, structural and functional alterations [5]. Malformations of the cranial base suggest associated anomalies of the brain (rhombencephalic region) and larynx during embryonal development [21].

Specific growth charts for CdCS, based on a multicentre study carried out on 374 patients from the United States, Italy, the United Kingdom and Australia, confirmed the existence of prenatal and postnatal growth retardation [22]. For all ages, median head circumference and weight are near or below the 2^nd ^and 5^th ^percentile, respectively. Height is less affected than weight from birth up to 2 years of age in both sexes. This trend continues until later in life, especially in males. The low weight may be attributed to feeding difficulties and gastroesophageal reflux, both of which are frequent in the first years of life [23]. On the other hand, the slender body shape of many adolescent and adult patients [5,9,14,24] may also be related to the syndrome.

The following features develop with age: the face becomes long and narrow (70.8%), the supra-orbital arch prominent (31.0%), the philtrum short (87.8%), the lower lip full (45.2%), dental malocclusion (open bite) (75.0%) (Fig. 1C,D), palpebral fissures tend to become horizontal (70.2%), divergent strabismus is frequent (44.7%), metacarpi (82.6%) and metatarsi (75.0%) are short resulting in small hands and feet, and prematurely grey hair may be observed (30.4%) [5,7-19,24-28] (percentages from the Italian CdCS Registry [19]).

Myopia and cataract have been reported. Hypersensitivity of the pupil to methacholine and resistance to mydriatics, probably due to a defect of the pupil dilator muscle, have also been described [29,30]. These features have also been found in four patients with Goldenhar's syndrome associated with CdCS [31,32]. Scoliosis, flat foot, pes varus, inguinal hernia and diastasis recti are frequent. Two patients with joint hyperextensibility, skin hyperelasticity and other features of Ehlers-Danlos syndrome [5], and one patient with both clinical manifestations of CdCS and Marfan syndrome have been reported [33]. A patient with a small deletion in 5p15.33 and phenotype suggesting Lujan-Fryns syndrome has been described [34].

Cryptorchidism, sometimes present at birth, is rare in adolescent patients. Sexual development is generally normal in both sexes. A single case of procreation in a CdCS patient (a mother and a daughter with the typical syndrome) has been reported [35].

With age, muscle hypotonia is replaced by hypertonia, and microcephaly becomes more evident. Convulsive crises are rare at all ages. Atrophy of the brainstem mainly involving the pons, cerebellum, median cerebellar peduncles and cerebellar white matter has been revealed by magnetic nuclear resonance imaging [36,37]. A CdCS child with an arachnoid cyst, causing triventricular hydrocephalus by obstruction of the aqueduct of Silvius, has been reported [38]. Metabolic anomalies have been described in CdCS patients: a defect in the synthesis of purine nucleotides (important neuromediators involved in brain development) [39,40] and clinical features associated with non-ketotic hyperglycinaemia, infantile spasms, hypsarrhythmia and brain heterotopia have been reported in a patient with a 5p deletion and typical CdCS [41].

### Developmental and behavioural profile

The limited data available about the psychomotor development indicated a severe psychomotor and mental retardation in all patients [5,25]. Prognosis is better for home-reared patients who underwent an early educational program [42-44]. Progress in verbal development is particularly slow [5,45]. Patients' ability to comprehend speech is better than their ability to communicate [46].

A study on psychomotor development was carried out on 91 patients from the Italian Registry [18,47], using the Denver Developmental Screening Test II (DDST II) [48]. This test showed the percentile distribution of patients on the basis of the age of achievement of developmental milestones [47]. A specific psychomotor development chart has been established. Data from the Italian series show that half of the patients walk by themselves at three years old and that all learn to walk later; with regard to the language, 25% of the children are able to make short sentences at 4.5 years old, 50% at 5.5 and almost all the children make short sentences before the age of 10; 50% of the patients feed themselves with a spoon at 3.5 years old and dress at 5 [19]. Although these patients have a range of severe developmental retardation, they can achieve many skills in childhood and continue to learn. This suggests that today's CdCS patients have a better outcome than those in the past [19].

CdCS children have mostly a gentle and affectionate personality. Hyperactivity is present in about 50% of patients and sometimes coexists with aggressiveness, which can be modified with adequate educational programs [5,10,42,49]. The behavioural profile of 27 patients studied by Cornish and Pigram [44] showed self-injury, repetitive movements, hypersensitivity to sounds, clumsiness and obsessive attachment to objects. Hyperactivity and distractibility seems specific to CdCS, if compared to Prader-Willi and Smith-Magenis syndromes [50]. A survey of the prevalence of stereotypy, self-injury and aggression in CdCS children and young adults has been recently carried out by Collins and Cornish [51]. A low level of object-directed behaviour may be an early precursor of hyperactivity, distractibility and stereotypy in older individuals [52]. Nevertheless, early educational interventions and the involvement of families and caregivers allow these behaviours to be improved [19,42].

## Aetiology

**Figure 2:**
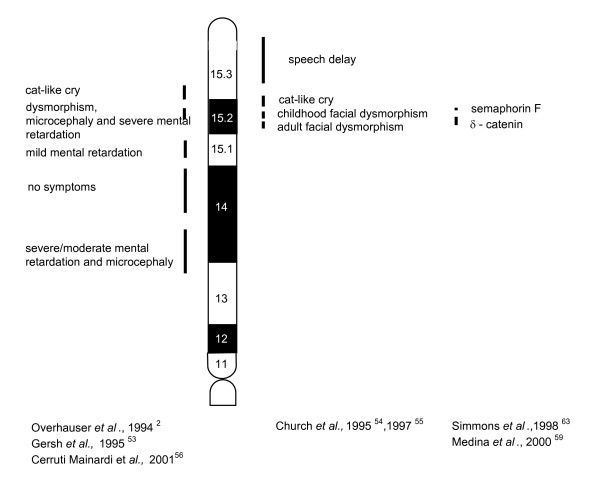
Phenotypic map of 5p. Vertical lines indicate the critical regions for the cry in p15.3, and for the other signs of Cri du Chat syndrome in p15.2. Vertical lines in p15.1, p14 and p13 refer to clinical symptoms reported in individual families with interstitial deletions.

The introduction of molecular cytogenetic analysis (Fluorescence *In Situ *Hybridisation, FISH) has allowed the cytogenetic and phenotypic map of 5p to be defined [2,53-56]. Analysis of 80 patients and 148 parents from the Italian Registry of CdCS revealed: a 5p terminal deletion (62 patients: 77.5%), an interstitial deletion (seven patients: 8.75%), a *de novo *translocation (four patients: 5%), a familial translocation (three patients: 3.75%), a mosaic with two rearranged cell lines (three patients: 3.75%) and a deletion originating from a paternal inversion (one patient: 1.25%). The breakpoints range from p13 to p15.2 (Fig. 2) [56]. This region contains a large number of repetitive sequences that may account for its instability [55,57]. Molecular analysis showed that the deleted chromosome is paternal in most cases: 20/25 (80%) [58], 10/12 (83.3%) [54], 55/61 (90.2%) [56].

The recent studies and observations of Italian patients suggest that partial aneusomy syndromes like CdCS result from abnormal gene dosage (haploinsufficiency) involving a large number of contiguous genes [3,55,56,59]. Other mechanisms, such as gene inactivation due to the position effect or rupture of a very large gene, have also been suggested [60].

A gene for chondrocalcinosis [61] and a gene for asthma [62] have been mapped to 5p15.2. The human Semaphorin F gene (*SEMAF*) covering at least 10% of this region has been cloned [63]. Due to its role in guiding axons or migrating neuronal precursors during cortical development in mice, it has been suggested that the *SEMAF *deletion may be responsible for some of the features of CdCS. Another gene, human **δ**-catenin (*CTNND2*), has also been mapped to 5p15.2 [59]. **δ**-catenin is a protein involved in cell motility and is expressed early in neuronal development. **δ**-catenin deletion seems to correlate with mental retardation in patients with a terminal deletion in this area [59]. **δ**-catenin knockout mice showed severe impairment of cognitive function, confirming the critical role of this gene in brain function [64].

The results of a recent study in CdCS patients suggest that haploinsufficiency of the telomerase reverse transcriptase (*hTERT*) gene, localised to 5p15.33, could contribute to the heterogeneous phenotype of CdCS. hTERT is the rate-limiting component for the telomerase activity that is essential for telomere-length maintenance and sustained cell proliferation [65].

## Genotype-phenotype correlation

Although CdCS is a well-defined clinical entity, individuals with 5p deletion show phenotypic and cytogenetic variability. A few studies, sometimes giving conflicting results, have been performed to correlate the clinical picture with the deletion size [5,24,56,66]. A more severe phenotype and cognitive impairment was reported to be associated with a larger deletion [10,67].

The fact that the phenotype is well recognisable, in spite of the variability in deletion size, has led to the hypothesis that a critical region causes the characteristic clinical picture when present in a hemizygous situation: Niebuhr located this region in a narrow area around 5p15.2 [5,68]. Such an assumption was supported by findings of individuals with a deletion that did not include 5p15.2, who either did not display the typical CdCS phenotype [69,70], or were completely normal [71].

Molecular-cytogenetic analysis allowed Overhauser *et al*. [2] and Gersh *et al*. [53] to identify two distinct regions, one for the typical cry in 5p15.3, and another for the other clinical characteristics in 5p15.2. Church *et al*. [54] distinguished several critical regions: a region for speech retardation, one for the typical cry, one for face dysmorphisms in childhood and one for face dysmorphisms in adulthood (Fig. 2).

A genotype-phenotype correlation study has been carried out in 80 patients from the Italian CdCS Registry. All of them underwent FISH analysis [56]. The results confirmed the importance of deletion of the critical region for manifestation of the CdCS clinical features. However, they also showed a clinical and cytogenetic variability and highlighted a correlation between clinical severity, and the size and type of deletion. In fact, in 62 patients with terminal deletion, the degree of severity (for microcephaly, dysmorphism and psychomotor retardation) has been demonstrated to vary between patients with a small deletion in 5p15.2 and 5p15.1, and patients with a larger deletion. The condition of patients with a deletion in 5p13 appeared particularly severe (Fig. 2).

The variability correlated with the type of deletion in patients with an interstitial deletion, unbalanced translocation resulting in 5p deletion, mosaicism and other rare rearrangements. The study of patients with an interstitial deletion and with a small terminal deletion has enabled the existence of two distinct critical regions (one for dysmorphisms, microcephaly and mental retardation in p15.2, and the other for the typical cry in p15.3) to be confirmed. Moreover, this study allowed the cry region defined by Overhauser *et al*. [2] to be narrowed distally and supported the hypothesis of a distinct region for speech retardation in p15.3 [54]. Furthermore, two patients who showed an interstitial deletion and a terminal deletion that did not include the critical region and did not show CdCS clinical features, confirmed that not all 5p deletions result in the CdCS phenotype [56,69,70].

In patients with an unbalanced translocation resulting in 5p deletion, the partial trisomy of the other involved chromosome may influence the clinical features, even if the CdCS phenotype prevails [72]. Three patients with mosaicism showed two rearranged cell lines: one with both cell lines deleted, the others with a deleted and a duplicated cell line. In the latter, the CdCS phenotype prevailed over the effect of the partial 5p trisomy present in part of the cells. The patient with the largest duplication had a mild clinical picture, suggesting compensation between deleted and duplicated cell lines [73]. Kitsiou *et al*. reported a patient with three cell lines in the same tissue: del 5p, dup 5p and a normal one. The mild phenotype in this patient could be mainly due to the normal cell line. However, the duplicated cell line may have contributed to the phenotype through the duplication of the critical CdCS region [73,74].

The deleted chromosome was mainly of paternal origin [54,56,58]: no phenotypic differences caused by imprinting effects were observed in the Italian group of patients [56].

The combination of FISH, comparative genome hybridisation (CGH) and cytogenetic analysis of a patient with dup5q/del5p confirmed that the characteristic cry was due to the deletion at 5p15.3 [75]. Recently Rossi *et al*. [76], using FISH analysis with bacterial artificial chromosome (BAC) clones in a patient without typical CdCS features, were able to correlate cat-like cry and mild mental retardation with a deletion in 5p15.31, 8.5 Mb away from the short arm telomere. Zhang *et al*. [77], by using array CGH, refined the CdC critical regions and confirmed the correlation between the severity of mental retardation and the deletion size and type. Using quantitative polymerase chain reaction (PCR), Wu *et al*. [78] narrowed the critical region for the cat-like cry to a short 640 Kb region and characterised three candidate genes in this region. Harvard *et al*. [79] found, in a subject with an autism spectrum disorder, a *de novo *cryptic microdeletion involving 5p15.2.

The identification of phenotypic subsets associated with specific deletions may be of great diagnostic and prognostic relevance. Furthermore, clinical examination combined with molecular analysis of the deletion results in a more personalised evaluation of the patients, which is useful for rehabilitative and educational programs [56].

## Diagnostic methods

The diagnosis is first of all clinical, based on typical characteristics such as facial dysmorphisms (facial gestalt), transverse flexion creases, hypotonia in combination with the peculiar cat-like cry. The first test to perform is karyotype analysis, which will confirm the diagnosis. In doubtful cases, when there is a conflict between the clinical suspicion and an apparently normal karyotype result, FISH analysis should be performed [19,34,56,76,80-83].

The importance of FISH for a precise diagnosis of 5p deletions must be emphasised. In the Italian series (80 patients), seven of the patients had not been correctly diagnosed by routine cytogenetics. FISH revealed that five of these patients had an interstitial deletion, one had a small terminal deletion and one had mosaicism [56]. Subtelomeric FISH allows 5p cryptic chromosomal rearrangements to be found [34,82]. Recent techniques, such as array CGH and quantitative PCR, mainly used for research purposes, allow a more precise definition of breakpoints and microrearrangements [77-79].

## Differential diagnosis

The clinical features of CdCS patients are not specific if considered separately but, if valued as a whole, they result in a distinct phenotype which, together with the peculiar cry, allows the diagnosis to be suspected at birth. Karyotype analysis of the peripheral blood will confirm the diagnosis.

In the mild cases that can escape the diagnosis or in older patients, it will be the clinical picture (and, above all, the voice that remains abnormal) and the psychomotor retardation that will lead to carrying out of cytogenetic and molecular cytogenetic analyses.

## Genetic counselling

The risk of recurrence is practically negligible for the cases of a *de novo *deletion, which are the most frequent. However, the possibility of gonadal mosaicism in one of the parents cannot be excluded, even if no recurrence has been reported up to now. It is higher for cases of balanced familial translocation. The reproductive risk for carriers of translocations involving 5p has been defined by evaluation of personal and reviewed data from 54 pedigrees [72]. The same study showed that the risk of unbalanced offspring (according to the pachytene configuration and 5p breakpoint localisation) ranged from 8.7% to 18.8%. The risk for male and female carriers was similar [72]. In these cases, prenatal diagnosis is appropriate.

## Antenatal diagnosis

Prenatal diagnosis by cytogenetic and molecular cytogenetic analyses has been reported in some cases with previous CdCS child, in which the syndrome resulted from a familial balanced translocation [84-88]. Prenatal diagnosis of *de novo *5p deletions is not frequent. In two cases it has been performed on the basis of a nonimmune foetal hydrops [89,90], and in another, on the basis of an abnormal ultrasound finding of isolated moderate bilateral ventriculomegaly [91]. Foetal choroid plexus cysts and/or abnormal maternal serum human chorionic gonadotropin (hCG) values in association with CdCS have been reported [92-95]. Chen *et al*. reported prenatal diagnosis of a foetus with 5p-mosaicism in a case involving advanced maternal age and carried out a review of the literature [88]. In their patient, the mosaic distal 5p deletion was found in association with sonographic markers such as microcephaly and cerebellar hypoplasia [88]. Prenatal diagnosis of the 5p deletion in association with Dandy-Walker syndrome and agenesis of the corpus callosum has been reported [96].

However, it should be noted that not all 5p deletions result in the CdCS phenotype: subjects with short terminal deletions in 5p15.3 may show only a mild or moderate psychomotor retardation [69,70,76,97,98]. Moreover, an interstitial and apparently unbalanced deletion in 5p14, detected by prenatal diagnosis indicated for advanced maternal age and traced through six individuals in three generations, resulted in a completely normal phenotype [71].

## Management

There is no specific treatment for CdCS as the cerebral damage resulting from the mutation occurs in the early stages of the embryonal development. Nevertheless, patients benefit from rehabilitative programs, which should be started as soon as possible and involve close collaboration with families, who must be supported psychologically. Moreover, it is important to give to the families updated information about the syndrome, also available through CdCS Support Groups.

Neonatal problems can generally be treated in neonatal pathology departments and intensive treatment is rarely necessary. Breast feeding is possible. For newborns with difficulties in suction and swallowing, physical therapy should start in the first weeks of life. If malformations are present, neonatologists and paediatricians should suggest diagnostic investigations and specialist examinations. It is important to highlight the risk of anaesthesiological problems (intubation difficulties) linked to larynx and epiglottis malformations [99,100]. Intubation difficulties were observed in three cases in the Italian series, but at an older age many patients underwent general anaesthesia without complications [19].

Early rehabilitation (physical therapy, psychomotricity, speech therapy) is recommended for the neurological problems such as psychomotor and speech retardation. As some patients have sensory-neural deafness and speech retardation, audiometric examination should be carried out on all CdCS children. All advised vaccinations are recommended.

Upbringing and rehabilitation are equally important for improvement of the social adaptation of the patients. Guidelines for treatment and follow-up have been reviewed elsewhere [17-19,101].

## Prognosis

After the first years of life, the survival expectation is high and morbidity is low. The mortality in the series studied by Niebuhr was about 10%, 75% of which occurred during the first months of life, and up to 90% within the first year [5]. Among the cases described in this study, three patients have lived to be over 50 years of age. Updated data have been reported in a recent study on the natural history of CdCS in a large series of Italian patients [19]. Recent improvements in management of patients with CdCS, with the application of rehabilitative programs, have led to increased psychomotor development, improved autonomy and better social adaptation [19].
